# Prevalence and Clinical Significance of Latent Brugada Syndrome in Atrial Fibrillation Patients Below 45 Years of Age

**DOI:** 10.3389/fcvm.2020.602536

**Published:** 2020-11-19

**Authors:** Ramadan Ghaleb, Matteo Anselmino, Luca Gaido, Stefano Quaranta, Carla Giustetto, Mohammed Kamal Salama, Ayman Salh, Marco Scaglione, Enas Fathy, Fiorenzo Gaita

**Affiliations:** ^1^Department of Cardiovascular Medicine, Aswan University Hospital, Aswan, Egypt; ^2^Division of Cardiology, Department of Medical Sciences, “Città della Salute e della Scienza di Torino” Hospital, University of Turin, Turin, Italy; ^3^Department of Cardiovascular Medicine, Kafr El-Sheikh University Hospital, Kafr El-Sheikh, Egypt; ^4^Department of Cardiovascular Medicine, Ain Shams University Hospital, Cairo, Egypt; ^5^Division of Cardiology, “Cardinal Massaia” Hospital, Asti, Italy

**Keywords:** sudden cardiac death, class 1 antiarrhythmic drugs, Brugada syndrome, atrial fibrillation, transcatheter ablation

## Abstract

**Aim:** This study aims to describe prevalence and clinical significance of latent Brugada syndrome (BrS) in a young population with atrial fibrillation (AF).

**Methods:** Between September 2015 and November 2017, among 111 AF patients below 45 years of age, those without pre-existing pathologies and/or known risk factors were selected for the study. Based on baseline 12-lead−24-h Holter electrocardiogram (ECG), previous class 1C antiarrhythmic drug therapy, or ajmaline testing, patients were stratified as latent type 1 BrS or not.

**Results:** Within the 78 enrolled patients, 13 (16.7%; group 1) revealed a type 1 BrS ECG pattern, while 65 (83.3%; group 2) did not. Mean age was 37 ± 8 vs. 35 ± 7 (*p* = 0.42), and males were 7 (54%) vs. 54 (83%) (*p* = 0.02) in the two groups, respectively. Family history of BrS was significantly more common within group 1 patients (2, 15% vs. 0; *p* = 0.03), and 4 (31%) patients experienced syncope in group 1 vs. 5 (8%) in group 2 (*p* = 0.02). After a mean follow-up of 42 ± 18 months from the index AF event, more than 80% of the patients, in both study groups, were in sinus rhythm.

**Conclusion:** In young patients with AF without pre-existing pathologies and/or known risk factors, latent BrS should be suspected. Syncope and a family history of BrS emerge as easily identifiable factors related to BrS. Long-term sinus rhythm maintenance appears satisfactory, either in the presence or not of BrS.

## Introduction

Brugada syndrome (BrS) is a genetic disease that accounts for ~20% of sudden cardiac death (SCD) cases in young, healthy adults with structurally normal hearts (1). BrS may be asymptomatic, or it may present with a variety of symptoms, such as syncope and palpitations. BrS is diagnosed in the presence of a coved ST-segment elevation ≥2 mm followed by a negative T wave in at least one right precordial lead positioned at the second, third, or fourth intercostal spaces, either spontaneously or after provocation by intravenous (IV) administration of class 1 antiarrhythmic drugs (AADs).

Approximately 20% of patients with BrS develop supraventricular arrhythmias—most commonly atrial fibrillation (AF) (2). The incidence of AF in patients with BrS has been reported to vary from 11 to 39% (3) and is considered to be an index of poor prognosis. Conversely, latent BrS has been reported in patients with new-onset AF (3–5).

The aim of the present study is to evaluate prevalence and influence on clinical management and outcomes of latent BrS in young patients with AF without pre-existing pathologies and/or known risk factors.

## Materials and Methods

### Study Population and Definitions

Between September 2015 and November 2017, 111 consecutive patients who presented at either of two centers in Italy (Città della Salute e Della Scienza Hospital, Turin and Cardinal Massaia Hospital, Asti) with AF at an age below 45 years were screened. Referral to the hospital was mainly due to AF management or previous indication of the physician in charge to transcatheter ablation. Out of 111 patients, the 78 (70%) not presenting pre-existing pathologies and/or known risk factors were enrolled.

The diagnosis of a BrS ECG pattern was established according to the criteria of the second consensus conference (1) and according to the expert consensus statement of the Heart Rhythm Society, European Heart Rhythm Association (EHRA), and Asia Pacific Heart Rhythm Society (APHRS) in 2013 (6). All patients were searched for any cause, other than BrS, that may explain ST-segment elevation (e.g., myocardial infarction, ventricular aneurysm, coronary artery spasm, Takotsubo cardiomyopathy, pericarditis, hyperkalemia, myocarditis, left bundle branch block, and pulmonary embolism). The study was performed in accordance with the latest Declaration of Helsinki principles, and patients provided informed consent to be included in the clinical data registry approved by the local Ethical Committee.

### Study Design and Groups

General cardiac examination, basal 12-lead ECG, and transthoracic echocardiography were performed in all patients. The occurrence of syncope and a family history of SCD, BrS, or AF in first-degree relatives were investigated. Pharmacological history with special concern for class 1C antiarrhythmic drugs such as flecainide or propafenone and follow-up ECGs performed during the treatment periods were recorded. In individuals with ECG patterns suggestive of BrS, 12-lead−24-h Holter ECG and class 1A AAD pharmacological testing with ajmaline were performed.

More in details, basal ECG tracing results were analyzed in all patients to evaluate the presence of a BrS ECG patterns (type 1, 2, or 3), either spontaneously or after the administration of class 1C AADs in at least one right precordial lead, including recordings from the second and third intercostal spaces (6). Additionally, the detection of rSr′ waves were described according to the criteria published by Chevalier et al. (7). In cases of type 2 or 3 BrS patterns, or doubtful interpretations, 12-lead−24-h Holter ECG was performed by placing leads V1 and V2 in the second intercostal space, and with V3 and V4 in the standard V1 and V2 positions in the fourth intercostal space, to identify periods of spontaneous type 1 pattern. ECGs were evaluated independently by two authors (E.F. and S.Q.) and, in case of divergent interpretation, by a third author (C.G.), blinded on patient's medical history.

Data related to the pharmacological drug therapy at the time of the first admission given either for cardioversion or as chronic therapy for rhythm control was analyzed. Patients who had ECG results suggestive of BrS and were under chronic therapy with class 1C AADs were not subjected to class 1A AAD testing, as the ECGs during the treatment period were considered comparable with the results of the pharmacological test. In case of ECG patterns suggestive of BrS (not related to chronic therapy with class 1C AADs) or strong clinical suspicion of BrS due to syncope and/or a family history of SCD/BrS, patients underwent pharmacological class 1A AAD testing with ajmaline. The drug was administered by slow intravenous infusion at a dose of 1 mg/kg over 5 min. Serial ECGs were recorded throughout the test. The test was terminated upon observation of a diagnostic type 1 BrS ECG pattern, premature ventricular beats/arrhythmias, or QRS widening >130% vs. baseline (1). The test was considered positive upon the appearance of a type 1 ECG pattern.

On this basis, patients were divided into two groups: AF and a type 1 BrS ECG pattern that occurred either spontaneously or in response to class 1 AAD therapy or ajmaline testing (group 1) and AF without BrS (group 2).

### Follow-Up

Follow-up data were collected by clinical evaluation or telephone contact to identify: new symptoms, such as syncope or aborted SCD; incidence of new BrS diagnosis (from other centers) in either patients or their first-degree relatives; and cardiovascular events. Arrhythmia recurrences, defined according to the European Society of Cardiology guidelines (8), were investigated through hospital records or follow-up Holter ECGs.

### Statistical Analysis

Continuous variables are reported as the mean ± standard deviation or median and interquartile range (IQR), depending on data distribution. Between-group comparisons were performed with the maximum-likelihood χ^2^ test or Fisher's exact test for categorical variables and Student's *t*-test for continuous variables. All probability values are two sided, with *P* < 0.05 considered to indicate a significant difference. Analyses were performed with SPSS version 25 (IBM, Armonk, NY, USA).

## Results

**Figure 1:**
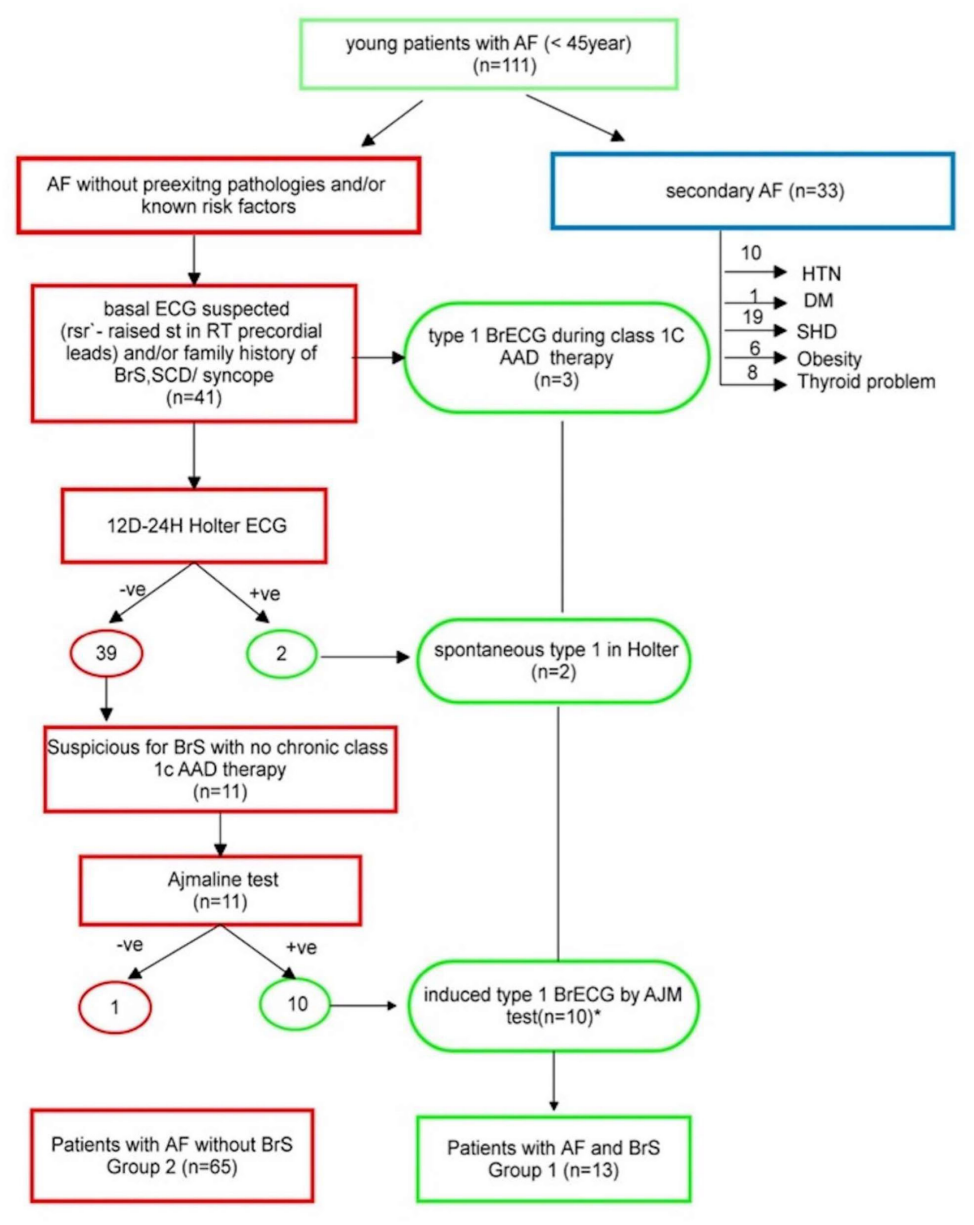
Study flowchart. ^*^including the two patients who are +ve in Holter. AAD, antiarrhythmic drug; AF, atrial fibrillation; DM, diabetes mellitus; HTN, hypertension; SCD, sudden cardiac death; SHD, structural heart disease; +ve, positive; –ve, negative.

In details, as illustrated in Figure 1, among the 111 screened patients, 78 (70%, mean age 35 ± 7 years) presented AF without pre-existing pathologies and/or known risk factors.

**Figure 2:**
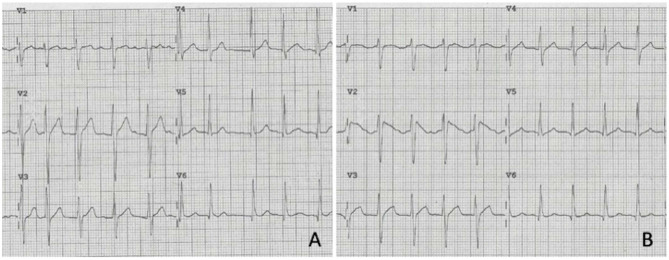
Precordial leads of the ECG of patient No. 13 showing atrial fibrillation with no suspicion for Brugada pattern. **(A)** At sinus rhythm restoration by IV Flecanide, type 1 Brugada ECG pattern emerged in V1–V2 **(B)**.

**Figure 3:**
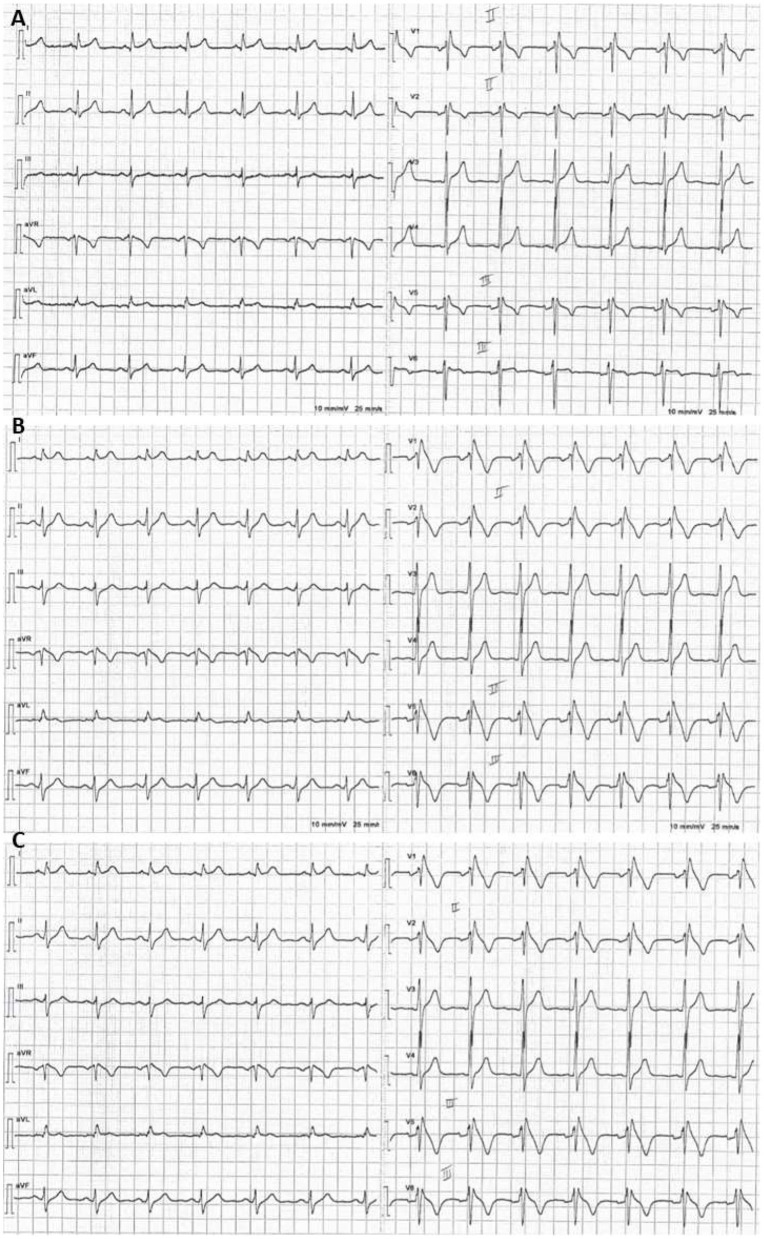
Example of a positive ajmaline test: basal ECG with V1–V2 in the 2nd intercostal space showing minimal r′. **(A)** Five minutes after starting intravenous infusion of ajmaline, a type 1 BrS ECG pattern emerged **(B)** and persisted over the following minute **(C)**.

Based on ECG assessment, three patients (patients 1, 7, and 13; Table 1) presented at least one ECG with a type 1 BrS ECG pattern during treatment with class 1C AADs. In details, in patient No. 1, the BrS ECG pattern was identified during chronic oral therapy with flecainide; in patient No. 7, the pattern was identified 3 h after IV administration of propafenone; and in patient No. 13, the pattern emerged after IV flecainide (Figure 2). Forty-one (52.6%) patients, instead, presented an ECG suggestive of BrS (either BrS ECG type 2 or 3 or rSr′) and underwent 12-lead−24-h Holter ECG recording. The latter identified a spontaneous type 1 BrS ECG pattern in two patients. A suggestive BrS pattern (see criteria in the “MATERIAL AND METHODS” section) emerged, instead, in 11 individuals, that therefore underwent ajmaline testing (Figure 3). In 10 (90.1%), a type 1 BrS ECG (patients 2–6 and 8–12, Table 1) was induced. Of the 37 (47.4%) patients who did not have an ECG suspicious for BrS, 34 (91.9%) were on chronic oral class 1C AAD therapy.

**Table 1:** Individual clinical characteristics of patients with atrial fibrillation and BrS type 1 ECG patterns (group 1).

**Patient no**.	**Age (years)**	**Sex**	**Family history of SCD**	**Family history of BrS**	**Syncope**	**Basal ECG**	**ED**	**EPS**	**ICD**	**Genetic test**	**TC ablation**	**TTT or AAD**
1	20	M	No	No	No	rSr′	AVRT	No	No	No	Yes	Beta blocker
2	23	M	No	No	Yes	rSr′	No	–ve	No	No	Yes	No
3	39	F	Yes	No	No	rSr′	No	No	No	No	Yes	Beta blocker
4	38	M	No	No	Yes	Type 2	SSS	+ve	Yes	+ve	Yes	HQ
5	45	F	No	Yes	No	Type 3	No	No	No	–ve	No	HQ
6	43	M	No	No	No	Type 3	No	No	No	–ve	No	No
7	42	M	No	No	Yes	Type 3	No	+ve	Yes	–ve	No	HQ
8	28	M	No	No	No	rSr′	No	No	No	No	Yes	Beta blocker
9	41	M	No	No	Yes	Type 2	No	No	No	No	No	HQ
10	40	F	No	No	No	Type 2	No	No	No	No	No	HQ
11	45	F	No	Yes	No	Type 3	No	No	No	+ve	No	HQ
12	43	F	No	No	No	Type 2	No	No	No	–ve	No	No
13	31	M	No	No	No	rSr′	No	No	No	No	Yes	HQ

*AAD, antiarrhythmic drug; AFL, atrial flutter; AVRT, atrioventricular nodal reentrant tachycardia; BBB, bundle branch block; BrS, Brugada syndrome; ECG, electrocardiogram; ED, electrical disturbance; EPS, electrophysiology study; F, female; HC, hydroquinidine; M, male; SCD, sudden cardiac death; SSS, sick sinus syndrome; TC, transcatheter*.

The enrolled population was, therefore, divided into two groups: patients with AF and a type 1 BrS ECG pattern that occurred either spontaneously or in response to class 1 AAD therapy or ajmaline testing (group 1; *n* = 13, 16.7%) and those with AF without BrS (group 2; *n* = 65, 83.3%). Baseline clinical characteristics, stratified by study group, are summarized in Table 2.

**Table 2:** Basic clinical characteristics of the study population, stratified by group.

**Patient** **characteristics**	**AF patients with BrS (group 1)**	**AF patients without BrS (group 2)**	***P*-value**
Mean age (years)	37 ± 8	35 ± 7	0.42
Male	7 (54%)	54 (83%)	*0.02*
Family history of BrS	2 (15%)	0	*0.03*
Family history of SCD	1 (8%)	1 (1.5%)	0.20
Family history of AF	0	7 (11%)	0.22
Syncope	4 (31%)	5 (8%)	0.02
Vagally mediated AF	2 (15%)	15 (58%)	0.01
Paroxysmal AF	13(100%)	54 (83%)	0.40
Persistent AF	0	12 (19%)	0.09
Ejection fraction (%)	60 ± 4	60 ± 6	0.68
Dilated LA (AP >40 mm)	2 (15%)	29 (45%)	0.05
PR (ms)	160 ± 35	158 ± 29	0.86
QRS (ms)	83 ± 8	94 ± 14	*0.01*
QTc (ms)	420 ± 25	399 ± 29	0.03
Early repolarization	1 (8%)	13 (20%)	0.28
Antiarrhythmic drugs^*^	6 (46%)	50 (77%)	0.02
Sotalol (class II)	0	15 (23%)	0.05
Class 1C	1 (8%)	39 (60%)	<0.01
Amiodarone (class III)	0	7 (11%)	0.21

*Fisher's exact test for categorical variables and Student's t-test for continuous variables; P < 0.05, considered to indicate a significant difference, in italics*.

*AF, atrial fibrillation; AP, anterior–posterior; BrS, Brugada syndrome; ECG, electrocardiogram; LA, left atrium; PAF, paroxysmal atrial fibrillation; SCD, sudden cardiac death*.

* *At the time of the first observation*.

Mean age was 37 ± 8 vs. 35 ± 7 years (*p* = 0.42), and 7 (54%) vs. 54 (83%) were males (*p* = 0.02) in the two groups, respectively. Family history of BrS was significantly more common within group 1 patients (2, 15% vs. 0; *p* = 0.03), while that of SCD and AF did not significantly differ (1, 8% vs. 1, 1.5%; *p* = 0.20 and 0 vs. 7, 11%; *p* = 0.22, respectively, in groups 1 and 2). Four (31%) patients had experienced syncope in group 1 vs. 5 (8%) in group 2 (*p* = 0.02). While PR interval (94 ± 14 vs. 83 ± 8 ms, *p* = 0.01) did not differ in the two groups (158 ± 29 vs. 160 ± 35 ms, *p* = 0.86), QTc (399 ± 29 vs. 420 ± 25 ms, *p* = 0.03) was longer in group 2 than in group 1 patients. In group 1, all patients suffered paroxysmal AF vs. 54 (83%) in group 2 (*p* = 0.40).

### AF Management and Outcome

Sixty-five patients, 13 (100%) and 56 (86%) in groups 1 and 2, respectively, were followed for a mean period of 42 ± 38 months from the index AF. Relevant clinical characteristics of all the 13 group 1 patients are listed in Table 1. Notably, three of these patients underwent electrophysiology study either due to spontaneous type 1 ECG patterns or syncope. Due to positive outcome, two of them underwent implantable cardioverter defibrillator (ICD) implantation. Six patients underwent genetic testing, while the remaining refused genetic analysis. In search of SCN5A and SN1B genetic variants and SCN10A single nucleotide polymorphisms (SNPs), two patients yielded positive results for a SCN5A mutation (c.611 + 1G>A) and a SNP in SCN10A, respectively.

Specifically concerning AF management, hydroquinidine therapy, based on previous experience of our group (3), was initiated as rhythm control strategy, in 11 patients. The two remaining patients did not experience further AF relapses after the index event and remained free from AADs. Due to inefficacy of or intolerance to hydroquinidine, transcatheter ablation was performed in six patients: pulmonary vein isolation (PVI) was performed in five; one patient, instead, underwent ablation of a concealed left lateral accessory pathway.

All group 2 patients underwent AF transcatheter ablation. Thirty-eight patients received PVI only, while 17 were additionally treated by cavotricuspid isthmus ablation, and 10, experiencing persistent AF, by complex fractionated atrial electrogram ablation and/or atrial linear lesions. Fifty-five (85%) patients were on AADs at the time of ablation: 4 were receiving amiodarone, 34 flecainide, 11 sotalol, 5 propafenone, and 1 quinidine.

In both groups, transcatheter ablation did not expose the patients to stroke, transient ischemic attack, or death.

At study end, six (86%) of the seven patients prescribed hydroquinidine in group 1 were in sinus rhythm; one patient, instead, continued suffering paroxysmal AF relapses (and refused transcatheter ablation). Out of the six patients who underwent TC ablation, five (83%) presented sinus rhythm, while one continued documenting paroxysmal AF. Overall, in group 1, 11 (84.6%) patients did not suffer atrial arrhythmia relapses. In group 2, instead, following transcatheter ablation, 47 (84%) presented sinus rhythm (21 with concomitant AADs), while 9 (16%) experienced AF recurrences (in seven cases paroxysmal, one persistent, and one permanent).

## Discussion

In this study, among 78 AF individuals of an age below 45 years without pre-existing pathologies and/or known risk factors, 13 (16.7%) had a type 1 BrS ECG pattern spontaneously (*n* = 2) and/or pharmacologically induced by class 1 AAD chronic oral therapy or testing (*n* = 11). This proportion is higher than what was reported previously (3–5) and suggests AF may plausibly relate to latent BrS. One reason may relate to the mean age of our study that, given the inclusion criteria of patients ≤45 years of age, was 37 ± 8 years, lower than that in other studies (4, 9, 10).

Early diagnosis of BrS in individuals with AF might enable timely application of life-saving clinical management protocols. Given that ECG changes in BrS are dynamic, a single basal ECG may be inconclusive. Presentation without a diagnostic ECG is reported in up to 60% of the subjects with BrS (5). In fact, to increase the probability of detecting type 1 ECG patterns, recording of 12-lead ECGs repeatedly in the same individual is recommended (11). In this study, for example, two patients had a spontaneous type 1 BrS ECG only at 12-lead−24-h Holter, supporting the emerging role of this technique for diagnosis and monitoring BrS patients, as well as for screening family members. Although the diagnostic role of class 1 AAD pharmacological testing for concealed BrS in patients with AF is not emphasized in the current European Society of Cardiology AF guidelines (8), it surely holds potential (4, 5). In the present population, the outcome of the response to class 1 AAD oral therapy or ajmaline testing enabled the identification of a non-negligible percentage of patients with BrS.

AF may surely represent an early, benign presentation of BrS. In a previous study, the prevalence of aborted SCD was 9% among 23 individuals with AF onset subsequent to the diagnosis of BrS, whereas 25 individuals who first presented with AF did not experience ventricular arrhythmias and instead had good long-term prognosis (3). In another study on 190 patients with lone AF, among 11 who were diagnosed with BrS, none had SCD, and three had VF events that were properly managed by ICDs (4).

Family history of BrS and/or of SCD is present in ~20–30% of the BrS population (12). Although most studies have not demonstrated a clear association between family history of SCD and VF events (13), in a Japanese registry, a family history of sudden death was an independent predictor of subsequent VF events (HR 3.28; 10). In our population, patients in group 1 reported a 15% of family history of BrS, significantly higher than that reported in lone AF patients without latent BrS. Similar figures have been reported in a study focused on AF patients without pre-existing pathologies and/or known risk factors (5), suggesting this aspect should methodically be investigated in young patients presenting with AF.

In addition, syncope is a known predictor of sudden cardiac arrest in patients with BrS (10). Approximately 20% of patients suffering sudden death due to BrS had at least one syncope episode prior to the fatal event (14). Group 1 patients presented, in fact, compared with group 2, a significantly higher prevalence of syncope, supporting the need for careful analysis of syncope in patients with AF. Although, none of group 2 patients had conduction delays, QTc interval was significantly prolonged compared with group 1 patients, suggesting the presence of an underlying channellopathy, as may be seen in SCN5A variant carriers.

Eventually, given the optimal therapeutic strategy for AF without pre-existing pathologies and/or known risk factors is not clearly documented, the present study highlights several relevant issues concerning rhythm management. Although HQ in BrS patients is mainly recommended for preventing ventricular arrhythmias, and may expose to an increased risk of sudden cardiac death in the general population (15), it has also been suggested, prescribed at monitored dosage and under defined clinical circumstances (e.g., avoiding dysionias), to be safe and effective also for the prevention atrial arrhythmias, and specifically AF (3, 16). In group 1, in fact, six patients achieved effective rhythm control by HQ; although generalization of the present finding is strongly limited by the small number of subjects involved, HQ, in our opinion, candidates as a reasonable alternative to more commonly prescribed AADs in this specific patient group. Moreover, transcatheter ablation surely achieves favorable results in the general population (17); however, little is known in BrS patients. In the present study, 46% of group 1 patients underwent transcatheter ablation, with an AF recurrence rate of 16% at study end. In a previous study, no instances of AF recurrences were observed in 13 of 14 BrS patients following PVI (18) and, in the experience by Conte et al. AF transcatheter ablation has also been shown to reduce inappropriate shocks in BrS implanted with an ICD (19). These evidences emphasize the role of transcatheter ablation for AF also in patients with BrS, supporting it represents a safe option in a population of patients in whom the use of traditional AADs is, in any case, limited.

### Limitations of the Study

Even though this study involved two centers, the sample population available was limited. The involvement of a larger number of hospital centers or a longer recruitment period would increase the population size and could improve result generalization and reliability of the statistical analyses. Additionally, ~10% of patients were lost to follow-up and therefore not included in the analysis.

## Conclusion

We identified a high prevalence (16.7%) of latent BrS in patients below 45 years of age presenting without pre-existing pathologies and/or known risk factors. Given the relevance of recognizing this condition, enabling tailored management of the SCD risk, spontaneous or pharmacologically induced ECG patterns, as well as clinical risk factors as family history or syncope, should methodologically be searched for. Awaiting larger clinical studies, although based on a limited sample size, the present study highlights, in young patients with AF and latent BrS, the favorable rhythm control outcomes of hydroquinidine therapy and, following inefficacy or intolerance, of transcatheter ablation.

## Data Availability Statement

The original contributions presented in the study are included in the article, further inquiries can be directed to the corresponding author/s.

## Ethics Statement

The studies involving human participants were reviewed and approved by Comitato Etico Città della Salute e della Scienza di Torino and Comitato Etico Cardinal Massaia Hospital, Asti. The patients/participants provided their written informed consent to participate in this study.

## Author Contributions

EF: data collection, follow up, analysis and interpretation, writing, and drafting. MA: analysis and interpretation, writing, and revision. LG and SQ: data collection and follow up. CG: design or conceptualization of the study, interpretation, and revision. MS: data collection and conceptualization of the study. AS: revision. MKS and RG: analysis, interpretation, and revision. FG: design or conceptualization of the study and revision. All authors contributed to the article and approved the submitted version.

## Conflict of Interest

The authors declare that the research was conducted in the absence of any commercial or financial relationships that could be construed as a potential conflict of interest.
